# Features of endometrial cancer in patients with ‘metabolically healthy’ versus ‘standard’ obesity: the decreasing frequency of metabolically healthy obesity

**DOI:** 10.4155/fso.15.68

**Published:** 2015-11-01

**Authors:** Lev M Berstein, Tatyana E Poroshina, Elena A Turkevich, Dmitry A Vasilyev, Alexandra N Baltrukova, Irina M Kovalenko, Igor V Berlev

**Affiliations:** 1 Laboratory of Oncoendocrinology, N.N. Petrov Research Institute of Oncology, St Petersburg 197758, Russia; 2 Department of Tumor Morphology, N.N. Petrov Research Institute of Oncology, St Petersburg 197758, Russia; 3 Division of Oncogynecology, N.N. Petrov Research Institute of Oncology, St Petersburg 197758, Russia

**Keywords:** endometrial cancer, metabolically healthy obese, obesity types, tumor features and subtypes

## Abstract

**Background:**

As endometrial cancer (EC) prevalence increases with obesity, we aimed to determine whether EC characteristics depend upon obesity type: ‘standard’ (SO) or ‘metabolically healthy obesity’ (MHO).

**Patients & methods:**

258 EC patients were included. Data on anthropometry, blood hormones, lipids and glucose, and tumor features were collected.

**Results:**

EC clinicopathologic characteristics and clinical stage correlate differently with BMI and obesity type. BMI is related inversely with tumor grade while SO patients are characterized by a more advanced clinical stage than those with MHO. Besides typical insulin resistance signs, EC patients with SO often display a higher serum leptin/adiponectin ratio compared with MHO patients. Historical data suggest a gradual increase in EC patient height and weight, and a decrease in MHO prevalence.

**Conclusion:**

It is currently unknown whether the latter observation reflects the evolution of EC, or obesity alongside the current epidemic. Regardless, the reduced MHO prevalence demonstrates the need for more intensive preventive measures aimed at obesity and obesity-associated conditions, including different EC subtypes.

## Abstract (lay abstract)

Worldwide prevalence of uterine body or endometrial cancer (EC) has increased notably. Recently, the WHO documented an obesity epidemic in several countries. Concurrently, many studies showed a high (37–66%) obesity rate in EC patients. There is currently no mention of a potentially distinctive correlation of EC with obesity types. In general, these types of obesity can be subdivided into ‘standard’ (SO; with metabolic disturbances, initially with insulin resistance signs) and ‘nonstandard’, or metabolically healthy obesity (MHO; without mentioned disturbances). The present paper concludes, first, that EC patients with SO (especially if BMI is ≥30) generally have more advanced tumor stage than patients with MHO. Second, in an EC group (2012–2014) a decrease in MHO frequency versus 1998–2000 group was observed. Altogether, this underlines the need for additional cancer preventive efforts in the obese female population.

**Figure 1.**
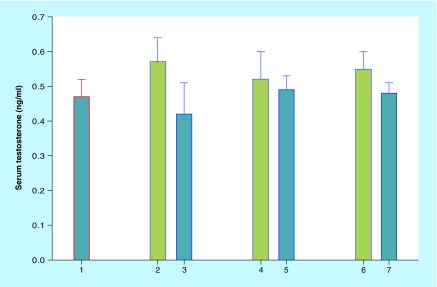
**Serum testosterone level (ng/ml; mean ± standard error) in endometrial cancer patients.** 1: Patients with BMI <25. 2, 4, 6: Patients of MHO (‘metabolically healthy obese’) group with BMI values, respectively, 25.0–29.9, ≥30.0 and ≥25.0. 3, 5, 7: Patients of SO (‘standard obesity’) group with BMI values, respectively, 25.0–29.9, ≥30.0 and ≥25.0.

**Figure 2.**
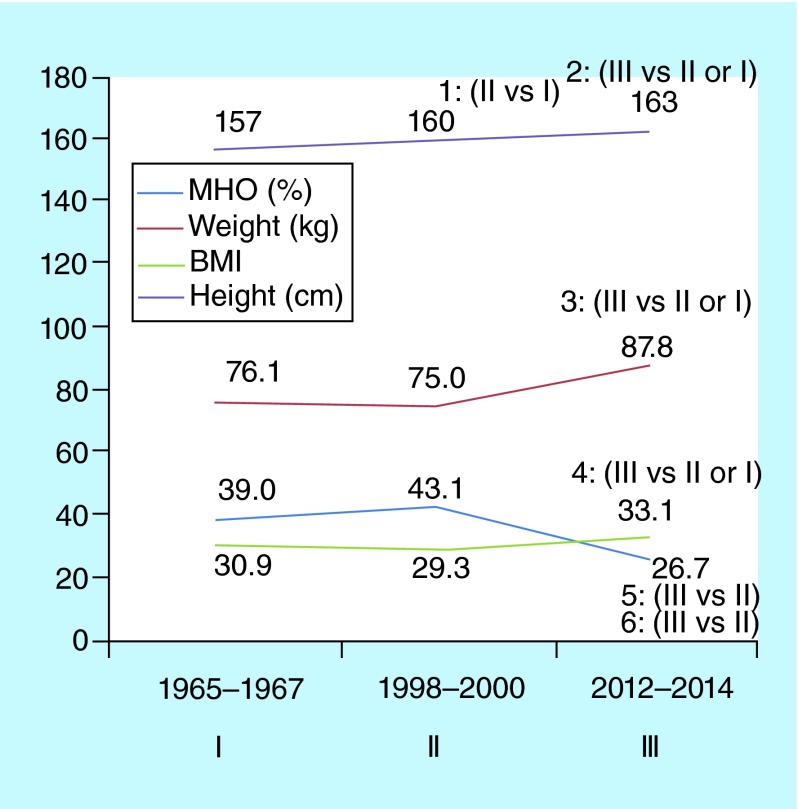
**Comparison of anthropometric parameter values and the frequency of ‘metabolically healthy obesity’ in endometrial cancer patients evaluated in 1965–1967 (I), 1998–2000 (II) and 2012–2014 (III).** p-value according to the Student *t*-test: 1: <0.02; 2: <0.01; 3: <0.01; 4: <0.01; 5: <0.05; 6: <0.02. Presented data on p-value are based each time on the comparison of two (and not multiple) groups. For example, p for height is calculated for the pair ‘Period II/Period I’ or ‘Period III/Period II’, and so on.

The prevalence of malignant tumors as a rule increases with age. Some of these tumors occur most frequently in menopausal women when there is commonly an increase in body mass. Recently, the WHO documented an obesity epidemic in several countries [1–3. While obesity is epidemic it is also highly heterogeneous [4,5]. This heterogeneity should be remembered while analyzing its epidemiology and impact on obesity-associated hormone-dependent tumors, including endometrial cancer (EC).

Worldwide prevalence for EC has increased notably [6,7]. Concurrently, several meta-analyses conducted in Caucasian populations showed a high (37–66%) obesity rate in EC patients [8–10]. The study of the prevalence and characteristics of EC and the correlation to obesity began long ago [11,12]. However, the data collected suggested discrepant opinions, especially on the association of obesity with EC features at diagnosis and the course of disease [13–17].

These discrepancies can be explained in part by the commonplace evaluation of BMI instead of body fat content [3], a lack of clarity in regards to the actual role of steroidal (estrogens, progesterone, testosterone) and nonsteroidal (mostly insulin, leptin, adiponectin) components in obesity–cancer associations; and, a very probable and gradual evolution of EC characteristics themselves. Of note, this last point is mentioned infrequently but may play a significant role as evinced herein [7,18]. In addition to some other frequently ignored factors, one should note the rather peculiar biology of EC (making it strikingly different from other hormone-dependent cancers, e.g., breast). Furthermore, there is no mention of a potentially distinctive correlation of EC with obesity phenotypes [4,5]. In general, these phenotypes can be subdivided into ‘standard’ and ‘nonstandard’ categories.

According to modern concepts of obesity, most of its negative effects are due to combined adiposopathy (fat tissue function impairment) and insulin resistance (IR). The above mentioned obesity categories are distinguished mainly on whether IR is present and participates as a factor leading to profound alterations in hormone-metabolic processes (‘standard’) or not (‘nonstandard’), while body fat content increases in both cases. The ‘nonstandard’ variant is often described as a ‘metabolically healthy obesity’ (MHO) state [19–21]. Mechanisms that could explain the more favorable metabolic profile of MHO individuals are poorly understood to date [21] and most aspects of how MHO relates to cancer development have yet to be studied [22–24], which also is true for EC patients [5,23].

As we reported previously, IR symptoms (independent of body fat content) correlate at the time of EC diagnosis with more advanced clinical stage and higher rates of local tumor invasion [25]. Whether this is true for obese patients without IR signs and associated hormone-metabolic features, remains unclear. It is important to stress here that the less favorable prognostic phenotype of treatment-naive EC is characterized commonly by higher HER-2/neu expression [26] and lower expression or mutation of the tumor suppressor *PTEN* [27] even though PTEN status remains a point of controversy [28]. Of note, there have been only a few studies on tumor PTEN expression in obese EC patients [29] where attempts to describe a correlation to obesity phenotypes were not made.

The current hospital based study was aimed at evaluating the association of BMI and obesity type (‘standard’ i.e., with IR signs, and ‘nonstandard’ or MHO) with important EC characteristics in treatment-naive patients studied in 1998–2000 and 2012–2014. In addition, the comparison of anthropometric parameters and the frequency of MHO in EC patients was made using data collected over the following periods: 1965–1967, 1998–2000 and 2012–2014.

## Patients & methods

A total of 258 EC patients were included on different stages of the study, which was approved by the local Ethics Committee and carried out in accordance with Declaration of Helsinki principles (2008 revision). With regards to the epidemiological design this was a ‘descriptive study’ where the source of population, that is patients with newly discovered EC, was all from the gynecological oncology clinic of N.N. Petrov Research Institute of Oncology. The usage of three time periods for patient evaluation (1965–1967; 1998–2000 and 2012–2014) was explained by the concentration of our group scientific interests on the problem of endometrial cancer just during those chronological phases of the last half-century and was justified by the presence of the same person (LMB) who participated in (1965–1967) or led (1998–2014) these studies.

The mean patient age was 60 years and this value was approximately the same when different time periods of patient data were compared. Data on histomorphological evaluation of the tumors are presented in the ‘Results’ section; in 2012–2014 around 77% of the tumors were endometrioid adenocarcinomas. Body mass (in kg) and height (cm) of patients were measured in the Institute facility for arriving patients, and BMI was calculated as bodyweight kg/height (m)^2^. The waist circumference was measured too. Correlation between BMI value and depth of tumor invasion (in mm), differentiation (grade) and disease stage was evaluated for all patients (see the scale for clinical stage evaluation in Table 1), while fasting serum insulin levels and insulin resistance index (HOMA-IR [30]), were evaluated in patients (n = 192) admitted in 1998–2000 and 2012–2014. A total and visceral fat content was evaluated in last group of patients indirectly using bioelectrical impedance analysis (Tanita BC-543 Body Composition Monitor, Tokyo, Japan; manufactured in 2011). Serum insulin, leptin, estradiol, estrone and testosterone levels were evaluated by ELISA using kits from DRG Instruments GmbH, Marburg, Germany, while adiponectin levels were determined with kits from DRG International Inc., Springfield Township, NJ, USA (with the day-to-day variance as a measure of analytical accuracy and parameter of quality control on the level 6.12, 6.43, 5.19, 5.74, 5.33 and 6.72%, respectively). Cholesterol, triglycerides and glucose in serum were evaluated by enzyme colorimetric assays using kits from Vector Best, Novosibirsk, Russia (day-to-day variance 3.69, 3.37 and 3.65%, respectively). Serum samples from venous blood were obtained after overnight fasting.

In 70 randomly selected patients from the 2012–2014 group an additional immunohistochemistry assay for tumor tissue PTEN (mouse monoclonal 6H2.1) and HER-2/neu (c-erbB-2; polyclonal A0485) expression was performed using Dako Company (Glostrup, Denmark) antibodies. Epitope retrieval was performed with heating buffer DAKO TRS S1699, pH 6.0; the warm-up time/heating time was 30 min. Antibodies were diluted 1:100 (PTEN) and 1:400 (HER-2/neu) by Dako antibody diluent. The incubation time was 30 min at 30°C. Dako Real EnVision, HRP, rabbit/mouse K5007 (PTEN) and Dako EnVision, HRP Labelled polymer, antirabbit K4002 (HER-2/neu) were used as secondary antibodies (incubation time 30 min, room temperature). The chromogen used was DAB in Dako Real Substrate buffer (all reagents from Dako Company, Glostrup, Denmark). See the ‘Results’ section for the criteria used to evaluate expression levels.

Patients admitted in 1998–2000 and 2012–2014 (192 pts total) were divided further into three groups based on BMI values: less than 25.0, 25.0–29.9 and ≥30.0. Patients with BMI value of ≥25.0 were evaluated additionally for obesity type (MHO or SO); see ‘Discussion’ section for explanation. Females who did not have a minimum three of the five following signs, based mostly on the criteria described in [31]: glucose tolerance impairment (fasting glucose ≥6.1 mmol/l), hypertriglyceridemia (≥1.7 mmol/l), increased waist circumference (≥88 cm), low serum high-density cholesterol level (≤1.3 mmol/l) and hypertension (≥130/85 mm Hg), were placed into the MHO group.

In patients with MHO and SO a comparative analysis of tumor characteristics, insulin level, insulin resistance index value (HOMA-IR calculated by the formula [glucose, mmol/l × insulin, μU/ml]/22.5]) and other parameters was performed based on the same principles as in BMI value-based groups. Additionally, data on the dynamics of anthropometric parameters and MHO prevalence in EC patients based on material collected by one of the authors (LMB) [32] for years 1965–1967 (n = 66), then collected in 1998–2000 and processed by Kvatchevskaya (n = 74) [25], and finally, based on information obtained by us in 2012–2014 (n = 118) were evaluated and compared. This complex and ‘extended’ study structure is explained by its early date of initiation. These chronological periods were chosen, as said in the beginning of this section, because of the interest of the authors toward the problem of endometrial cancer and acquisition of the data in those time periods. This ‘periodical’ interest helped us assess whether endometrial cancer and patients with this tumor are same now as they were several decades ago. Obtaining informed consent was not mandatory for patients whose personal data were not recorded; therefore, it was sufficient to obtain an approval of the Local Ethics Committee to determine if an investigation corresponded to principles later formulated by the 2008 Declaration of Helsinki revision, which we strictly followed in the course of the study.

Statistical analysis was performed for this descriptive study using parametric and nonparametric methods. SigmaPlot for Windows and Statistica 8.0 software was used. Descriptive statistics were utilized to report demographic characteristics and anthropometrics. Variables were tested for normality using the Kolmogorov-Smirnov test. The comparison of hormone-metabolic values between different groups (M ± SE) was performed by student's *t*-test. PTEN and HER-2/neu expression and clinical stage data were compared by χ^2^ test with one degree of freedom and Fisher's exact one-tailed test. The critical p-value throughout the whole study was 0.05. The generalization of these findings (based on the studied sample) to a larger population will be the task for future studies.

## Results

Higher BMI values were associated in EC patients with an increase in fasting serum insulin level and insulin resistance index. Additionally, the shift to more prognostically favorable variants of tumor differentiation, in agreement with data obtained previously [13,33], along with a tendency toward less evident myometrial invasion was revealed in patients with BMI ≥30. At the same time, no connection between BMI and clinical stage of cancer was found in EC patients (Table 1).

Although a probable shift with years in the distribution of EC morphologic variants and their correlation to anthropometry parameters will be considered separately in another publication, we would like to present some preliminary generalizations, which already can be made based on our data. In years 2012–2014 77.2% of EC patients were diagnosed with endometrioid adenocarcinoma, while in the groups distinguished on the basis of BMI value (<25.0, 25.0–29.9 and ≥30.0) this variant of the tumor was revealed in 70.6, 71.4 and 81.2% of cases, accordingly. In MHO and SO patients endometrioid adenocarcinoma was diagnosed in 77.7 and 78.5%, respectively. In other words, an increase in BMI can be considered a ‘modifying’ factor in regard of tumor morphology, which was described previously [13,16], while obesity type cannot. Therefore, it was justified to examine whether EC patients belonging to SO and MHO groups and studied in 1998–2000 and 2012–2014 differed in other tumor characteristics and perhaps related hormonal characteristics.

A partial response to this question can be found in Table 2, which also contains respective data on nonobese (BMI <25.0) EC patients without metabolic changes. First, patients belonging to MHO group are younger and, as expected, have lower insulin resistance index and insulinemia values. Second, which is more related to the main goals of this study, although there was no difference in EC differentiation (clinical grade) and invasion depth between patients belonging to SO and MHO groups, SO patients were characterized by more advanced tumor stage, which was most evident in BMI ≥30 group (Table 2). Of note, when insulin resistance index (HOMA-IR) as well as serum insulin, leptin and adiponectin levels were compared in EC patients, it was found that SO and MHO groups can be most effectively distinguished by HOMA-IR value and the ratio of leptin/adiponectin in serum. At the same time, serum leptin and adiponectin concentrations alone failed to demonstrate notable distinctions between these groups, especially when BMI value was ≥30 (Table 3).

Serum estradiol and estrone levels were slightly higher in SO patients compared with patients with MHO (in relative values for BMI ≥25 group: 100 and 95.6%, estradiol and 100 and 96.0%, estrone), although in absolute values this difference was not statistically significant (data are not presented). The same conclusion is valid for blood testosterone levels, although EC patients with MHO, in contrast to estrogenemia, displayed values somewhat higher than in SO patients (Figure 1). Evaluation of total and visceral body fat content did not reveal a statistically significant increase in SO group compared with MHO patients except a total body fat value in patients with BMI 25.0–29.9: SO 42.43 ± 0.79% versus 40.06 ± 0.78% in MHO, p = 0.04 and besides data in group of EC patients with BMI ≥25.0 (SO vs MHO: total fat content 46.13 ± 0.59% and 42.06 ± 0.83%, p < 0.01; visceral fat 13.68 ± 0.38% and 11.07 ± 0.83%, accordingly, p < 0.01).

Immunohistochemistry of EC markers (HER-2/neu and PTEN) was performed to correlate their expression with BMI, and SO versus MHO. Immunohistochemical evaluation of the tumor suppressor protein PTEN and the oncoprotein HER-2/neu in EC samples (n = 70) revealed a U-shaped correlation between BMI and PTEN expression: the weakest expression was found in patients with a BMI between 25.0–29.9. Tumor HER-2/neu expression had an inverse relationship to BMI values: minimal expression in patients with a BMI ≥30 (Table 4). At the same time, separate analysis of tumor tissue samples obtained from patients with distinct obesity phenotypes revealed the difference between groups. When obesity type was considered, a significant positive trend was found between BMI and PTEN expression in MHO group that does not exist in SO patients. In addition, tumors in MHO patients having BMI values between 25.0–29.9 displayed significantly lower HER-2/neu expression compared with nonobese patients (BMI <25.0), which also was not evident in tumors from SO patients. This demonstrates that besides a difference in clinical stage (Table 2) there are additional distinctions that exist at the tumor level between EC patients defined by different obesity phenotypes (Table 4).

Finally, the comparison of anthropometric features and MHO prevalence values in EC patients diagnosed in late 1960s (Period I), 1998–2000 (Period II) and 2012–2014 (Period III) discovered changes of studied parameters in the interval between Periods II and III. This observation manifested itself mostly in the form of pronounced body mass and height increases while the MHO frequency in Period III was lower (Figure 2). This was especially noted in patients having a BMI ≥30 (not shown).

## Discussion

There is evidence of an increase in the incidence of EC [6,7], which makes it not only a medical but also a social problem paralleling a similar situation in breast cancer [34]. The social significance of EC is explained largely by its relation to obesity, which has grown into a global epidemic [1,2]. Many researchers view obesity as an important contributor to the increase in the number of obesity-associated malignancies, including EC [9–10,35], with some researchers convinced that obesity is a cause of EC (see [36]). EC patients as a rule are not losing weight, rather they are gaining weight. Indeed, most patients have been overweight or obese long before the diagnosis of endometrial cancer is made. Therefore, there is stronger reason to believe that BMI excess or obesity participate in shaping features of this cancer and not vice versa*.* Too frequently, however, there is a disregard with respect to the heterogeneous nature of obesity that influences many clinically relevant problems, including the problem of cancer in general and EC in particular. As a consequence, the notion that obesity is a heterogeneous condition [5] and EC-associated factor formed the basis of this study.

Coming to summation, we should first mention that the authors of this paper quite intentionally diverged from an accepted BMI-based obesity definition. As we know, according to WHO classification BMI ≥25 is considered as overweight and BMI ≥30 as obese. Nevertheless, in this investigation we included provisionally into the latter group the patients with both BMI values (≥30 and 25.0–29.9). The aim here was to examine how BMI value, on the one hand, and belonging to the MHO or SO group, on the other hand, is associated with clinical features of EC. Also, we wished to see whether there is marked discrepancy in this regard between what is defined by the terms ‘obesity’ and ‘excessive bodyweight’. For a better overall understanding of the problem it is necessary to proffer several words expounding on the difference between ‘standard obesity’ and the notion of ‘metabolic syndrome’. Although the role of metabolic syndrome in the development of EC has been studied and undoubtedly is important [18,25,36], one should note that BMI currently is not included in its official definition [37]. In addition, it is noteworthy that two types of EC have been discussed for several decades [13,38]. Current thought has these two types of EC characterized by a distinction in their etiology and risk factors [39], although such a conclusion has been questioned. Consequently, this leads to discussion of whether known causal factors are different for type I and type II EC or not [8,39]. Further, this brings to the forefront a discussion as to whether obesity plays the same role in both EC types [6,8,10,16,33]. On this basis, additional study of the problem and related subjects is warranted.

Thus, it is the strength of present study that obesity was not perceived as a singular entity. By separating obesity into MHO and SO groups, we revealed its different aspects in EC patients. Besides confirming a reverse correlation between BMI and tumor grade (Table 1), it allowed us to ascertain distinctions in certain tumor characteristics between patients with SO and MHO, including disease stage (Table 2) and tumor-related protein expression (Table 4). Another strong point of this study is its ‘historical/chronological view’, which helped to discover the dynamics of anthropometrical characteristics and MHO frequency in ЕС patients, covering a time span of nearly 50 years (Figure 2). One could suppose that a gradual increase in the height of EC patients is just another confirmation of the acceleration phenomenon [40] according to which the 1910–1915 generation (at the end of 1960s they reached 55–60 years) might have lesser height than 1955–1960 generation (currently 55–60). At the same time, there is an evident trend toward increased body mass, which most probably reflects the ongoing development of the obesity epidemic [1,2] and correlates with the increases in EC incidence [6,10,41].

This study also has some disadvantages or limitations; for example, we have omitted temporarily an analysis of EC subclassification based on tumor morphology and genetics [8,39,42]. As already mentioned, the relationship between EC morphology and obesity type (SO or MHO) is a matter for additional analysis since we completely understand that tumor morphology and subtype classification can change with years in different populations of patients [7]. Besides these points, while the study was hospital based and the sample size is relatively small (circa 260 patients), we need to stress that no special selection of the patients was made.

Another point for discussion lies in the nature of method used for body fat content determination. Although the method used (bioimpedance measurement) is indirect, it sufficed to fulfill the study objectives as the results of SO and MHO groups comparison appear to be quite accurate (see the ‘Results’ section). The usage of more sophisticated and simultaneously more expensive methods, like, for example, dual-energy x-ray absorptiometry and computed tomography in future studies will allow for comparative studies in this area to demonstrate suitability of bioimpedance under certain conditions [43] and to confirm received data.

The lack of conventional (common) definitions and criteria for MHO [5,21,44] seems to be more important. According to the opinion of some experts the MHO state could be relatively stable over a 20‐year perspective [45], and therefore, the term ‘healthy’ for this condition is justified only partly [46]. In addition, in the 1960s insulin levels and HOMA-IR values could not be determined, and therefore retrospective data were not available for EC patients from the early period. Nevertheless, as the most evident decrease in MHO prevalence was observed between 1998–2000 and 2012–2014 (Figure 2), in other words, when the signs of insulin resistance were taken into consideration, there is a basis to consider this observation as important and reflecting not only on the evolution of obesity but the evolution of EC itself too [7,18,47], see additionally below.

The progression to a more advanced EC clinical stage in patients with SO (Table 2) might be promoted by higher proportions of total and visceral body fat content, severe insulin resistance, higher serum leptin/adiponectin ratio and probably the mild prevalence of estrogenemia but not testosteronemia as compared with patients with MHO (Tables 1–3 & Figure 1). Here we briefly mention the following supportive observations. First of all, leptin and adiponectin receptor density in EC tumor tissue correlates directly for leptin and inversely for adiponectin receptors, with regional lymph nodes involvement [48,49]. On the other hand, total serum testosterone levels tend to be higher in patients with EC [50] as it occurs in postmenopausal women with developing insulin resistance [51]. This is contrary to men where the resistance to insulin is more often the sign of low androgen level [52]. Meanwhile, there is no data to date on testosterone levels in patients with MHO and our preliminary results that unexpectedly indicated mildly lower testosteronemia in patients with SO compared with patients with MHO (Figure 1) deserve further verification.

No less than one third of patients admitted in 2012–2014 had signs of Type 2 diabetes mellitus (DM2) discovered due to evaluation of the case histories or revealed during glucose tolerance test. Accordingly, the issue whether the combination of different variants of obesity, SO or, understandably much less frequent, MHO, and DM2 has any ‘specific’ influence on tumor biology in EC patients warrants special study, since to date this problem was studied only as applied to obesity *en masse* [13,53] and was concentrated so far mostly on peripheral regulatory pathways. Furthermore, most of these studies were done without evaluation of PTEN and HER-2/neu expression in endometrial tumor tissue. This provides a topic for further comparative studies in EC patients with or without DM2 as we have shown altered expression of these markers among the obesity subtypes, SO versus MHO, in EC patients.

Of note, although the tumor suppressor protein PTEN expression was mentioned just above in the context of tumor tissue, in mouse models where additional copies of PTEN are expressed, the mice have a longer life span, lower frequency of spontaneous tumors and an increase in brown adipose tissue [54]. According to published data, which are not completely consistent, the quantitative and functional characteristics of brown adipose tissue may be used as an indicator of a predisposition to obesity [55–57] and one of the markers of its heterogeneity [5,58]. This aspect of the problem also has not been studied in EC patients, although it undoubtedly presents scientific and practical interest and deserves attention. The understanding of importance of distinctive obesity phenotypes and their role in EC pathogenesis and clinical course can lead also to better utilization and implementation of treatments derived from adipose stem cells and factors secreted by them [59].

## Conclusion

The main results of this study are summarized in Tables 1–4 & Figures 1 & 2. Based on these data one can jump to the following conclusions. First, some endometrial cancer characteristics, such as differentiation, depth of myometrial invasion, clinical stage and expression of PTEN and HER-2/neu by tumor tissue are correlated to a variable degree with BMI value and obesity type (‘metabolically healthy obesity’ or ‘standard obesity’). In particular, it is rather well known [13,33] that there is better tumor differentiation in patients with increased BMI (Table 1), while, after ‘obesity partition’, the disease stage appears to be more advanced in EC patients with combination of SO and BMI ≥30 in contrast to patients with MHO and same BMI value (Table 2). Second, we compared historical data that provide evidence of progressive change in mean height and weight of endometrial cancer patients. These parameters are gradually increasing over time while, on the other hand, MHO is now encountered more rarely in the contemporary EC population (Figure 2). Therefore, the actual and anticipated increase in EC incidence may depend not only on the fast growth of the obesity epidemic, but also on changes in obesity structure and, presumably, in white/brown adipose tissue interrelations. As a whole, this may require reviewing and changing certain measures aimed at the prevention of obesity and obesity-associated conditions including uterine body cancer.

**Table 1.** **Features of the tumor and insulinemia in endometrial cancer patients (group of 2012–2014) depending on BMI.**

**BMI grades**	**Age (years)**	**BMI (cond.un.)**	**Differentiation (points^†^)**	**Invasion (mm)**	**Clinical stage (points^‡^)**	**Insulin (μU/ml)**	**HOMA-IR (cond.un.)**
<25 (n = 17)	59.4 ± 2.7	22.7 ± 0.3^*^	2.07 ± 0.18^***^	10.00 ± 1.51	2.66 ± 0.36	10.66 ± 1.07^*^	2.85 ± 0.37^*^
25.0–29.9 (n = 28)	61.0 ± 1.9	28.5 ± 0.2^*^,^**^	1.78 ± 0.13	9.33 ± 1.81	2.56 ± 0.32	18.51 ± 1.61^*^,^**^	5.27 ± 0.54^*^,^**^
≥30 (n = 73)	61.1 ± 1.0	37.3 ± 0.7^*^,^**^	1.65 ± 0.09^***^	7.68 ± 0.82	2.48 ± 0.14	21.10 ± 1.14^*^,^**^	6.91 ± 0.50^*^,^**^

^*,**^p < 0.01; ^***^p < 0.05; n = number of patients.

^†^G1 – 1 point, G2 – 2 points, G3 – 3 points.

^‡^1a – 1 point, 1b – 2 points а, 1c – 2.5 points, 2a – 3 points, 2b – 4 points, 3a – 5 points, 3b – 6 points, 4 – 7 points.

Cond.un.: Conditional unit; HOMA-IR – insulin resistance index.

**Table 2.** **Features of the tumor and insulinemia in endometrial cancer patients (joint group of 1998–2000 and 2012–2014) depending on whether they belong to ‘standard’ or ‘metabolically healthy’ obesity subgroups.**

**BMI grades, obesity types**	**Age (years)**	**BMI (cond.un.)**	**Differentiation (points)**	**Invasion (mm)**	**Clinical stage (points)**	**Insulin (μU/ml)**	**HOMA-IR (cond.un.)**
<25 (n = 33)	56.8 ± 1.6	22.43 ± 0.17	2.16 ± 0.12	6.52 ± 0.58	2.31 ± 0.19	9.79 ± 0.78	2.43 ± 0.17
25.0–29.9 (n = 55)							
SO (n = 26)	61.5 ± 1.7	28.25 ± 0.11	1.75 ± 0.15	7.47 ± 1.58	2.80 ± 0.32	18.38 ± 1.48	5.51 ± 0.57
		p < 0.01			χ^2^ 1.96 (p = 0.16)	p < 0.02	p < 0.01
MHO (n = 29)	57.5 ± 1.3	27.45 ± 0.19	1.76 ± 0.09	8.41 ± 0.95	2.43 ± 0.21	13.30 ± 0.95	3.30 ± 0.28
≥30 (n = 104)							
SO (n = 81)	60.80 ± 0.95	36.68 ± 0.62	1.72 ± 0.05	6.69 ± 0.83	2.53 ± 0.12	21.32 ± 1.18	6.86 ± 0.34
					p < 0.04	p < 0.05	p < 0.01
MHO (n = 23)	55.80 ± 1.65	35.88 ± 1.21	1.65 ± 0.12	6.47 ± 0.92	2.17 ± 0.12	18.1 ± 1.10	4.25 ± 0.39
≥25 (n = 159)							
SO (n = 107)	60.9 ± 0.65	34.70 ± 0.52	1.73 ± 0.05	6.88 ± 0.63	2.60 ± 0.12	20.6 ± 0.64	6.54 ± 0.28
		p < 0.01			p = 0.06	p < 0.01	p < 0.01
MHO (n = 52)	56.8 ± 1.02	31.12 ± 0.52	1.71 ± 0.07	7.55 ± 0.96	2.31 ± 0.12	15.4 ± 0.65	3.71 ± 0.21

See notes in Table 1 and section ‘Patients & methods’.

p-values are given with the aim to compare respective data in SO and MHO groups.

Cond.un: Conditional unit; MHO: Metabolically healthy; SO: Standard.

**Table 3.** **Comparison of leptinemia, adiponectinemia and insulinemia levels in endometrial cancer patients (group of 2012–2014) depending on BMI value and obesity type.**

**BMI grades and types of obesity**	**BMI (cond.un.)**	**L (ng/ml)**	**A (μg/ml)**	**Ratio L/A (cond.un.)**	**Insulin (μU/ml)**	**HOMA-IR (cond.un.)**
<25 (n = 17)	22.71 ± 0.30	5.23 ± 0.77	83.98 ± 13.43	0.11 ± 0.04	10.66 ± 1.07	2.85 ± 0.37
25.0 – 29.9 (n = 28)						
SO (n = 13)	29.01 ± 0.19	24.11 ± 4.98	39.44 ± 9.84	0.69 ± 0.14	21.89 ± 2.79	6.79 ± 0.98
		p = 0.11	p < 0.02	p > 0.02	p = 0.06	p < 0.02
MHO (n = 15)	28.03 ± 0.35	14.55 ± 1.68	75.92 ± 10.72	0.29 ± 0.09	15.80 ± 1.61	4.05 ± 0.38
≥30 (n = 73)						
SO (n = 61)	37.76 ± 0.75	33.71 ± 3.14	50.35 ± 3.66	0.91 ± 0.16	21.62 ± 1.27	7.37 ± 0.56
		p = 0.09		p = 0.07	p > 0.2	p < 0.02
MHO (n = 12)	34.95 ± 1.53	25.59 ± 3.84	49.05 ± 5.42	0.57 ± 0.10	18.40 ± 2.41	4.55 ± 0.64
≥25 (n = 101)						
SO (n = 74)	36.2 ± 0.66	31.88 ± 2.75	48.41 ± 3.49	0.87 ± 0.14	21.60 ± 0.81	7.28 ± 0.28
		p < 0.01	p < 0.05	p < 0.01	p < 0.01	p < 0.01
MHO (n = 27)	31.12 ± 0.52	19.20 ± 2.22	64.60 ± 7.18	0.41 ± 0.07	17.00 ± 1.03	4.27 ± 0.35

A: Adiponectin; Cond.un: Conditional unit; HOMA-IR: Insulin resistance index; L: Leptin; MHO: Metabolically healthy; n: Number of patients; SO: Standard.

**Table 4.** **PTEN и HER-2/neu expression in endometrial cancer tissue (immunohistochemical analysis).**

**BMI grades, number of cases**	**PTEN^†^**	**HER-2/neu^‡^**	**BMI (cond.un.)**
In patients with different BMI values:			
– <25.0 (n = 10)	0.550 ± 0.157	1.333 ± 0.167	22.82 ± 0.34
– 25.0–29.9 (n = 19)	0.417 ± 0.109	1.105 ± 0.201	28.45 ± 0.27
– ≥25.0 (n = 60)	0.602 ± 0.060	0.867 ± 0.102	34.09 ± 0.80
– >30.0 (n = 41)	0.683 ± 0.069	0.756 ± 0.115	36.71 ± 0.91
In patients with the signs of MHO:			
– <25,0, without MHO (n = 10)	0.550 ± 0.157	1.333 ± 0.167^3,4^	22.82 ± 0.34
– 25.0–29.9 (n = 10)	0.400 ± 0.145^1^	0.900 ± 0.314^3^	27.98 ± 0.39
– ≥25.0 (n = 18)	0.555 ± 0.114	0.777 ± 0.207	30.99 ± 1.23
– ≥30.0 (n = 8)	0.750 ± 0.164^1^	0.625 ± 0.263^4^	34.75 ± 2.10
In patients with the signs of SO:			
– <25.0; without SO (n = 10)	0.550 ± 0.157	1.333 ± 0.167^5,6^	22.82 ± 0.34
– 25.0–29.9 (n = 9)	0.438 ± 0.176^2^	1.333 ± 0.236^5^	28.97 ± 0.25
– ≥25.0 (n = 42)	0.622 ± 0.072	0.905 ± 0.117	35.42 ± 0.95
– ≥30.0 (n = 33)	0.667 ± 0.077^2^	0.788 ± 0.129^6^	37.18 ± 1.02

χ^2^ values: ^1)^ 3.60 (р = 0.057); ^2)^ 1.40 (р = 0.237); ^3)^ 4.56 (p = 0.032); ^4)^ 5.88 (р = 0.015); ^5)^ 1.06 (p = 0.30); ^6)^ 5.13 (p = 0.023). See the ‘Patients & methods’ section.

^†^PTEN expression in points (0; 0.5; 1); Pearson χ^2^ coefficient (χ^2^ with one degree of freedom) was calculated on the basis of the incidence of 1.0 reactions versus <1.0 reactions.

^‡^HER-2/neu expression in points (0; 1; 2; 3), while Pearson χ^2^ coefficient (χ^2^ with one degree of freedom) was calculated on the basis of the incidence of ≥1.0 reactions versus 0 reactions.

Cond.un.: Conditional unit; MHO: Metabolically healthy; SO: Standard.

**Executive summary**

Obesity can be divided into ‘standard obesity’ (SO; characterized by insulin resistance and associated hormone-metabolic changes) and nonstandard (‘metabolically healthy obesity’ [MHO]); this classification seems to be justified for the area of endometrial cancer (EC) research, although the optimal MHO definitions and criteria are still to be discussed.
EC patients with SO (especially if BMI is ≥30) generally have more advanced disease and a tendency to higher HER-2/neu expression in tumor tissue in contrast to patients with MHO.
EC patients with SO are characterized by some additional hormonal factors including a marked increase in the ratio of serum leptin/adiponectin and an ‘unexpected’ tendency to mildly lower testosteronemia compared with patients with MHO.
The analysis of anthropometric characteristics of EC patients over the last 40–50 years in the same hospital discovered a gradual increase of height, weight and BMI in this population. In EC group of 2012–2014 a decrease in MHO frequency was observed. As a whole, this can be considered as a marker of the shifts in obesity epidemic parameters, both quantitative as well as qualitative, which also could influence other, gradually accumulating, manifestations of endometrial carcinoma evolution.
Heterogeneity of obesity in combination with growing heterogeneity of endometrial cancer (evident, e.g., in the change of the ratio [7] and number [60] of EC subtypes) is a factor in favor of the development of additional preventive and control measures in accord with the title of the recent publication in this area: ‘Catch it before it kills…’ [61].
